# Drug-Checking with Molecularly Imprinted Polymers: Addressing Xylazine-Contaminated Fentanyl

**DOI:** 10.1021/acssensors.5c01931

**Published:** 2025-08-18

**Authors:** Ramiro Marroquin-Garcia, Gil van Wissen, Rocio Arreguin-Campos, Gabriela Goraşov, Rob van Osch, Thomas. J. Cleij, Kasper Eersels, Bart van Grinsven, Hanne Diliën

**Affiliations:** † Sensor Engineering Department, Faculty of Science and Engineering, 5211 Maastricht University, 6200 MD Maastricht, The Netherlands; ‡ Zuyderland Medical Center, 6419 PC Heerlen, The Netherlands

**Keywords:** biomimetic sensor, xylazine, fentanyl, opioid, imprinted polymers, rapid test

## Abstract

Xylazine adulterated Fentanyl is considered one of the deadliest drug threats ever faced in the United States. However, due to the complexity of the illegal drug supply, xylazine detection in the frame of drug-checking (harm reduction) remains an unsolved challenge, one which has cost numerous lives across North America. In this work, we present two sensor platforms for detecting xylazine in drug-checking applications. Both platforms are based on molecularly imprinted polymers (MIPs) using vinyl phosphonic acid as the main monomer. By combining the MIPs with thermometric and colorimetric readouts, we achieved xylazine detection in a wide concentration range, relevant in real-world scenarios. First, the heat-transfer method (HTM) allowed xylazine detection in the low concentration range within 15 min per concentration, with a limit of detection (LoD) of 16.5 ng/mL (75 nM). Second, a dye displacement assay employing methylene blue provided rapid (<5 min), visible detection with an LoD > 76 μg/mL-five times lower than the average concentration reported for street samples. Additionally, the colorimetric sensor exhibited potential for detecting various unwanted xylazine analogues, which could potentially act as fentanyl adulterants in the future. Most importantly, the colorimetric sensor consistently detected xylazine in a highly complex, street-like fentanyl sample containing five common interferents. The synergy of the proposed sensors offers a promising alternative for centralized and on-site xylazine screening in harm reduction settings.

## Graphical abstract

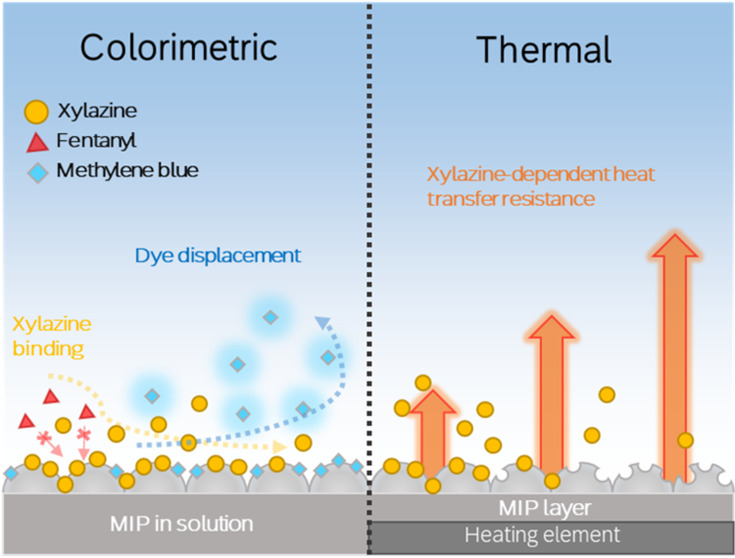

Fentanyl is one of the deadliest drug threats ever faced by the United States.^1^ Generally prescribed as an analgesic, fentanyl is 50 times stronger than heroin and 100 times stronger than morphine.^2^ Its presence in the US illegal drug supply started in 2013, and since then, overdose-related deaths have steadily increased, as 2 mg of pure fentanyl can be lethal.^3^ In 2021, the Drug Enforcement Administration (DEA) issued a public safety alert regarding a new fentanyl mixture containing the veterinary tranquilizer xylazine.^4^ This mixture, also referred to as “tranq” or “dope”, is responsible for a staggering 276% increase in fentanyl-involved deaths between 2019 and 2022.^5^ This increase is attributed to xylazine’s ability to suppress the effect of antioverdose drugs such as naloxone.^6^ Furthermore, prolonged xylazine use is strongly associated with necrotic skin lesions, which can increase the risk of limb amputation.^7^

Xylazine is an α-2 receptor agonist used as a sedative and muscle relaxant in some animals.^8^ This drug is believed to enhance the effects of fentanyl while reducing production costs, resulting in one of the most severe opioid crises in North America.^9^ Therefore, government agencies and public health organizations have proposed several harm reduction strategies to combat such an epidemic.^10^ These strategies aim to reduce the negative health consequences associated with drug consumption, including improving access to drug-checking technologies.^11^

Due to the complex composition of the illegal fentanyl supply, containing up to 8 different compounds, drug checking is considered one of the most important harm reduction strategies. In this case, spectroscopy methods and rapid test strips detect the presence of xylazine in street samples.^12,13^ However, these methods are often expensive, require trained personnel, and lack adequate selectivity and sensitivity, leading to false-positive results, hindering their on-site applicability.^14^ To address these limitations, we propose two molecularly imprinted polymers (MIPs) sensing platforms coupled with a thermometric and optical transducer for routine (drug-checking facilities) and on-site applications (rapid test). MIPs offer high specificity, adaptability, portability, and relatively low production cost, making them potential candidates for next-generation drug-checking technologies.

The combination of MIPs with thermometric transducers, such as the heat-transfer method (HTM), has demonstrated tremendous potential for the detection of small molecules.^15–17^ HTM translates the binding events on the MIPs surface into an increase in the system’s heat transfer resistance. It is due to the straightforward working principle and its relatively low cost that we propose the HTM-MIPs sensor platform to be used in centralized screening facilities.^18^

To further address the xylazine epidemic, this study also explores the use of MIPs within a colorimetric assay to enable in situ monitoring of this adulterant. The monomer employed in the MIPs hereby reported possesses a central role in this test. Vinyl phosphonic acid (VPA) is a diprotic acid with a similar hydrogen-bonding capacity as other commonly used monomers (such as methacrylic acid or MAA)^19^ but with an increased ability to bind positively charged molecules. This could translate into a MIP with excellent capability of capturing cationic dyes, which is exploited in this study for the development of the colorimetric rapid test aiming to support combat against the ongoing opioid crisis.

## Materials and Methods

All chemicals were used as received unless indicated otherwise. Xylazine free base (99%) was obtained from Bachem. Xylazine hydrochloride (99%), vinyl phosphonic acid (VPA, 97%), ethylene glycol dimethacrylate (EGDMA, 99%), 2,2′-Azobis (2-methylpropionitrile) (AIBN, 98%), 1,2-dichlorobenzene (HPLC grade), caffeine, and lidocaine hydrochloride monohydrate were obtained from Merck. Tizanidine hydrochloride (98%) and Clonidine hydrochloride (98%) were obtained from TCI chemicals. p-iodo-Clonidine hydrochloride (98%) was obtained from Cayman Chemicals. The stabilizer present in EGDMA was removed by running it through a basic alumina column. Methylene blue (Microscopy grade) was obtained from VWR. Fentanyl citrate solution was obtained from Hameln Pharma. Absolute methanol and acetic acid were obtained from Biosolve B.V. Double-sided adhesive carbon tape (nonwoven fabric base) was obtained from Nisshin EM Co., Ltd.

### Synthesis of Xylazine Molecularly Imprinted Polymers

The materials were prepared in triplicate via bulk free-radical polymerization at 65 °C (oil bath) for 12 h in 5 mL pressure vials. The preparation of the prepolymerization mixtures is as follows: VPA (345.7 mg, 3.2 mmol, 2 equiv) was directly weighed in the pressure vial. Followed by the addition of xylazine free base (352.54 mg, 1.6 mmol, 1 equiv), AIBN (55 mg, 0.33 mmol), and EGDMA (1208 mL, 6.4 mmol, 4 equiv). Before the vial was closed with the pressure septum, 3.6 mL of 1,2DClB was added. The NIP material was prepared as described but in the absence of xylazine. The vials were then sonicated for 30 min until all the components were completely dissolved. Before the polymerization, the homogeneous solutions were degassed using a strong stream of dry argon for 5 min. Polymerization started by placing the vials in the preheated oil bath for 12 h. After this time, the monoliths were removed from the vial and ground using a Fritsch Planetary Micro Mill Pulverisette7 premium line (3 cycles, 300 rpm, 3 min, and 5 zirconia balls *d*: 10 mm). The unreacted monomers and template were extracted from both MIP and NIP powders by Soxhlet extraction using a 1:1 v/v methanol:acetic acid solution and then pure methanol until no xylazine was detected in the solvent. Finally, the powders were dried at 65 °C for 24 h before their use.

### Xylazine Hydrochloride Rebinding Experiments in Aqueous Solutions

Rebinding of the xylazine hydrochloride was evaluated on the three MIP and NIP batches at the high concentration range (200–700 μM) in deionized water (DI water). In short, 6 × 20 mg (±0.1 mg) of both MIP and NIP were weighed in individual 12 mL glass vials (6 MIP-6 NIP). To each couple of MIP-NIP vials was added 4 mL of the desired xylazine concentration was added. The samples were then horizontally shaken at 180 rpm for 30 min. After this, the mixture was filtered by using a 0.22 μm PTFE syringe filter. The residual xylazine absorbance was measured from 350 to 200 nm after adequate dilution (0.5 mL filtrate-2 mL DI water) using a ultraviolet-visible (UV–vis) spectrophotometer. The binding isotherm was constructed by calculating the average xylazine free concentration in solution (*C*_f_) at 212 nm and plotted against the average template binding of the three batches (see eq 1).

1 $$S_{b}=\frac{s\mathrm{tarting}\;\mathrm{concentration}\;\left(\mu M\right)-C_{f}\;\left(\mu M\right)}{\mathrm{sample}\;\mathrm{weight}\;\left(g\right)}\times \mathrm{mL}\;\mathrm{of}\;\mathrm{solution}$$

### Carbon Tape Modified Functional Layer Fabrication

The MIP-NIP functionalized layers were prepared using commercially available scanning electron microscopy double-sided adhesive carbon tape.^16^ The tape specimens were cut (1 × 1 cm^2^) and adhered to an aluminum–magnesium (AlMg3, max 3 wt % Mg) circular disk (area: 1 cm^2^). Immobilization of the MIP and NIP particles was conducted by pressing the tape-aluminum disk onto a small quantity of powder placed on a Petri dish. Gentle taping and repressing were applied to ensure full coverage. After this, loosely bound particles were removed using water and then air-dried for 5 days before use.

### Heat-Transfer Method (HTM) Rebinding

The thermal setup was used as a slightly modified version of the one described elsewhere in the literature.^15–17,20^ In short, the functional layer is placed between a polycarbonate flow cell with an internal volume of 110 μL and a copper block sample holder. Temperature control (*T*_1_ = 37 °C) of the functional layer is provided via the copper block using a thermocouple, a power resistor, and a proportional-integral-derivative (P–I–D) controller (10–8–0.1). This temperature is applied through the functional layer to the liquid inside the flow cell. The flow cell temperature is continuously monitored using a second thermocouple (*T*_2_). The setup is completed by filling the flow cell with DI water. After stabilizing the temperature for 30 min, xylazine solutions of fixed concentrations (100 μM or 50 nM, depending on the test) are flushed (0.25 mL/min) inside the flow cell for 6 min and stabilized (0 mL/min) for 25 min before a new addition. Importantly, the continuous addition of xylazine with fixed concentration (100 μM or 50 nM) is here considered to be additive; that is, each injection contributed cumulatively to the total analyte concentration. Specifically, seven successive injections of the 100 μM solution resulted in a total analyzed concentration of 700 μM, while six injections of the 50 nM solution produced a final cumulative concentration of 300 nM.^21^ Furthermore, the binding isotherms were constructed from the thermal data, as reported in the past for MIPs, where effect size (ES) corresponds to the ratio of the change in temperature for each xylazine injection over the baseline temperature of pure DI water (see eq 2).

2 $$\mathrm{ES}\;\left(\%\right)=\frac{T_{\mathrm{water}}-T_{\mathrm{xylazine}}}{T_{\mathrm{water}}}\times 100$$

### Dye Rebinding and Loading

The dye rebinding proceeded similarly as described for xylazine hydrochloride but using 10 mg (±0.1 mg) of MIP-NIP powders. The binding isotherm was constructed similarly but using the residual absorbance at λ_max_ = 663 nm and adequate dilution of the filtered samples. For the dye loading, 700 mg of MIP powder was incubated with 100 mL (1 mM; in DI water) of MB for 2.5 h at 180 rpm. After this, the powder was placed in a Buchner funnel pore size (10–15 μm) and washed with 25 L of water to remove the MB excess. The final powder was dried at 65 °C before its use.

### Dose–Response Dye Displacement Assay, Selectivity, and Real Sample Analysis

Four mg of dye-loaded MIPs were weighed in 11 mL vials and then incubated with the desired concentrations of xylazine hydrochloride, selectivity/interferant compound, and the real samples (see Table S2) for 5 min. After this, the samples were filtered using a 0.22 μm PFTE filter and finally analyzed using a UV–vis spectrophotometer in the range 700–200 nm. Real sample was prepared using commercially available fentanyl citrate (50 μg/mL) solution containing 3.5 mg/mL NaCl and pH ∼ 7.4 according to the manufacturer’s data. The effect of the matrix was evaluated by incubating 4 mg of dye-loaded MIPs with an aqueous solution containing only 3.5 mg/mL NaCl and 50 μg/mL citrate monobasic (pH was adjusted to 7.4 using 0.01 M NaOH). The absorbance from this test was subtracted from that of the real sample. All tests were conducted in triplicate, and the dye maximum absorbance value was averaged.

## Results and Discussion

### MIP-NIP Evaluation in Aqueous Xylazine Solutions

The chemical composition of the polymer was studied by using FTIR spectroscopy. The presence of VPA signals (*e*.*g*., P–O–H, P–OH, and P=O) as well as C=O bonds from the EGDMA corroborates their incorporation in the polymer (See Figure S1). Furthermore, the performance of the 3 batches (MIP-NIP) was first evaluated using UV–vis spectroscopy. After incubating the polymers with the desired xylazine concentration solutions, the samples were filtered, and the remaining concentration (*C*_f_) and xylazine binding (*S*_b_) were calculated by using a calibration curve. It is clear from the results (see Figure 1) that a higher affinity toward xylazine was observed for the MIP when compared to the NIP; this is illustrated by the higher *S*_b_ and lower *C*_f_. Additionally, MIPs displayed a maximum *S*_b‑max_ of 86 μmol/g, whereas the NIPs *S*_b‑max_ was only 60 μmol/g. These binding results are considerably higher than those previously observed for MAA-based MIPs, which corroborates the advantageous effect of using the diprotic VPA monomer instead of MAA.^19^ Most importantly, due to the low standard deviation, the data suggest a good synthesis reproducibility and low interbatch variability. The latter is crucial to produce reliable sensors with an optimal performance. Finally, using the fitted allometric data (dotted lines), an imprinting factor-binding analyte MIP/binding analyte NIP-IF = 1.8 was calculated at 100 μM, which corroborates the presence of imprinted cavities in the MIP.^22^

**Figure 1:**
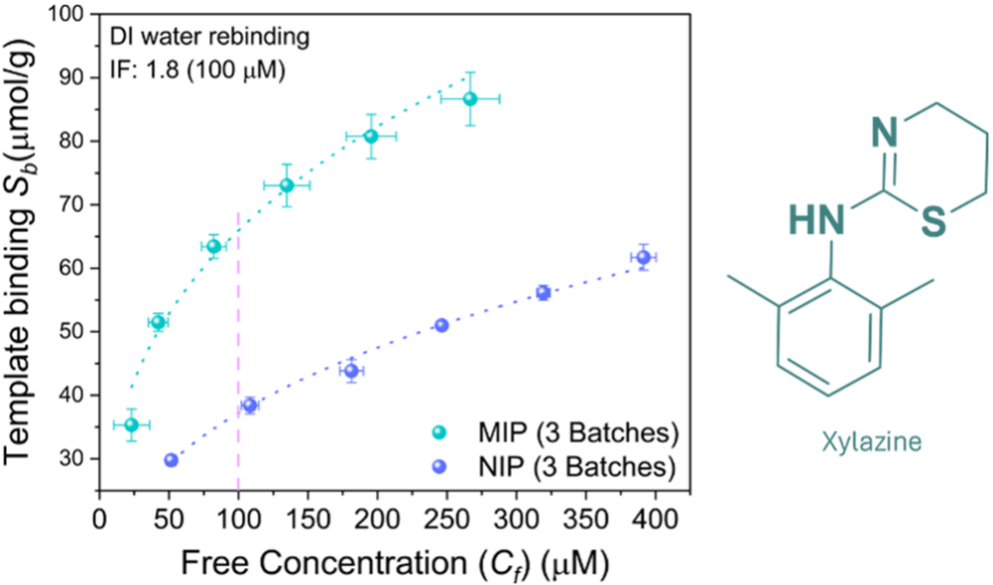
Xylazine chemical structure and binding isotherm constructed from the MIP-NIP UV–vis data after 30 min of rebinding in D.I water. The dotted lines represent the allometric data fit.

Although UV–vis spectroscopy is relatively fast and low cost, its main limitation is its high limit of detection (LoD), usually in the millimolar range for xylazine. To circumvent this limitation, we propose HTM as an alternative readout due to its potential to detect analytes at low concentrations (nM).^23^ To compare the results with the UV–vis, HTM measurements were first conducted using a fixed xylazine concentration of 100 μM, followed by a separate measurement using a lower concentration of 50 nM. In both cases, the measurements were considered additive, that is, each injection contributed cumulatively to the total analyte concentration.^15,17^ Specifically, seven successive injections of the 100 μM solution resulted in a total analyzed concentration of 700 μM, while six injections of the 50 nM solution produced a final cumulative concentration of 300 nM (Figure 2left,right, respectively). As seen from the binding isotherm at high concentration (Figure 2left), the materials display a binding profile similar to the one observed in the UV–vis (Figure 1). Both MIP and NIP exhibited a steady binding behavior over the whole range. However, such binding is considerably higher for the MIP, which displayed a maximum effect size (ES_max_) (see eq 2) of 1.2, which is twice the maximum observed in the NIP. Moreover, a LoD = 60 μM was estimated at the interception of the fitted line (green) and the 3σ noise values (dotted pink). Furthermore, when analyzing the materials in the low concentration range (Figure 2right), a similar trend was observed. In this case, a linear response was present after exposing the MIP to the xylazine solutions, giving an ES_max_ ∼ 0.9% at 300 nM. In contrast, the nonspecific binding response (represented by the NIP) remains constant (ES = 0.1%) during the first three injections. After this, the response increased linearly to half of the MIPs’ response ES_max_ ∼ 0.45% at 300 nM. In this test, the MIPs exhibited a LoD ∼ 75 nM, which is translated into 16.54 ng/mL, considering the flow cell volume of 110 μL. This value is 1 order of magnitude higher than the lowest reported one for xylazine electrochemical detection (4.5 nM).^24^ It is worth mentioning that such concentration cannot be analyzed in the UV–vis after the rebinding due to a lack of absorbance signal.

**Figure 2:**
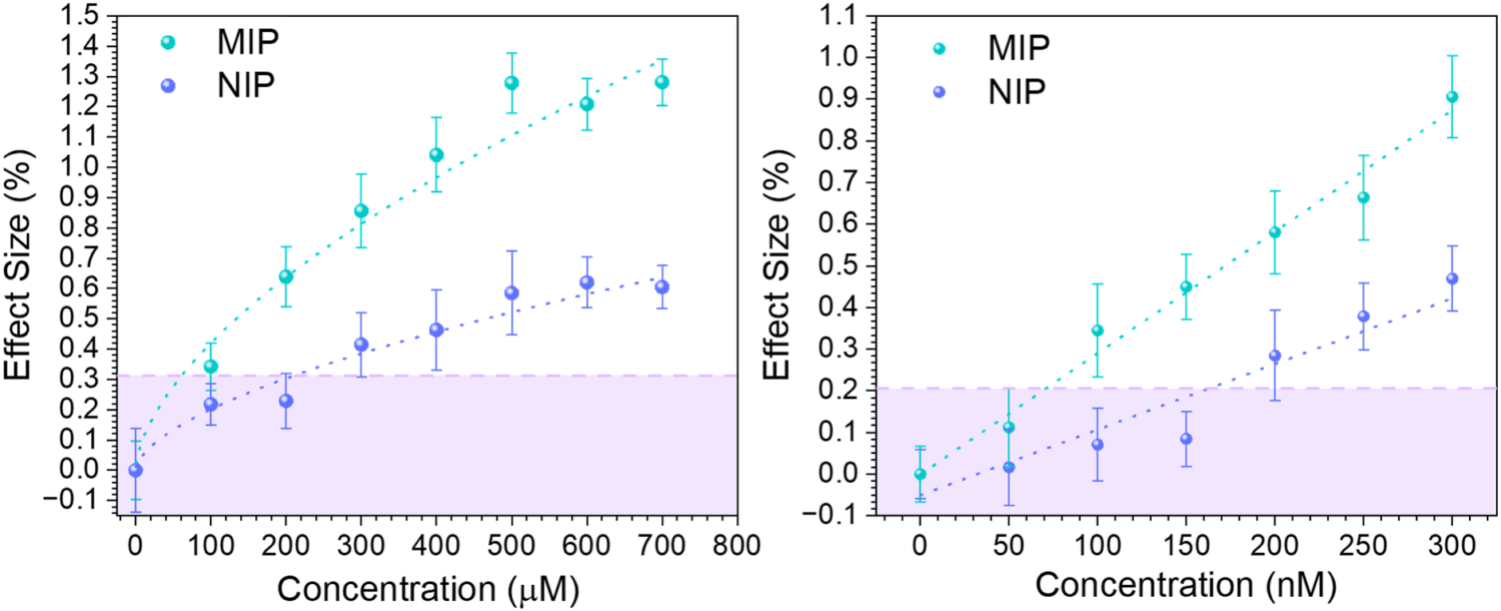
HTM xylazine binding isotherm at high concentrations (100–700 mM) (left) and low concentrations (50–300 nM) (right). Dotted lines (green and purple) correspond to the thermal data allometric fit, and the pink dotted line is the y-noise 3σ.

From these results, it is evident that measuring the same MIP-NIP system at different xylazine concentrations (100 μM or 50 nM) yields two different LoD’s: 60 μM and 75 nM, respectively.

In theory, analyzing higher xylazine concentrations (*e.g*., 100 μM) should produce a more pronounced thermal response when compared to lower concentrations, such as 50 nM. However, previous studies have suggested that the molecular cavities in MIPs are more selective and strongly occupied when exposed to low concentrations of the template. In contrast, high template concentrations tend to increase nonspecific binding, where the analyte is weakly and nonselectively associated with the polymer.

This difference in binding behavior explains the observed disparity in HTM LoD’s. When the 50 nM solution was measured (Figure 2right), specific binding sites are saturated in a stable manner, leading to a consistent thermal effect size of approximately 0.9%. Conversely, the 100 mM solution promotes more nonspecific interactions. These are less stable and do not block the surface as effectively, resulting in a slightly higher thermal effect (∼1.3%), despite the higher xylazine concentration.

The HTM’s response toward a wide variety of analytes, such as molecules, macromolecules, and even whole-cell targets, has been extensively studied.^15,25–29^ In the specific case of HTM coupled MIPs sensors, the thermal response arises from the selective binding of target molecules within the nanopores of the material. This event blocks heat flux and generates a measurable thermal response, a mechanism commonly referred to as the “pore blocking model”.^30^ To illustrate this effect, we estimated the number of xylazine molecules bound to the polymer at the initial injection of 100 μM in the HTM (see Supporting Information, (SI) for details). This corresponds to approximately 5.87 × 10^18^ molecules. While accurately quantifying binding at these early and subsequent stages remains challenging, the continuous increase in thermal response (1.3 and 0.9 %; see Figure 2left,right) suggests progressive target binding over time.

Comparable thermal responses have also been observed with covalently attached molecules such as thiols (*e.g*., 11-Mercaptoundecanoic acid) on gold substrates. Interestingly, such an effect was comparable to that observed for biofilm formation and cell layer attachment.^31^ However, in these latter examples, significantly fewer macromolecules or cells are typically required to produce a similar, or often greater, thermal response^25–29,32,33^ Notably, HTM LoDs correlate well with the values obtained from established electrochemical techniques such as impedance spectroscopy.^31,34^

In synthesis, these results highlight the potential of our HTM-MIP sensor for the detection of xylazine over a broad concentration range and in a relatively low time (15 min per concentration). However, to advance toward real-world applications, two critical factors need to be addressed. First, variation in the room temperature outside laboratory conditions can have a major impact on the sensor’s performance. To mitigate this, we propose the use of insulated chambers^35^ or portable cooling units, which can promote a stable internal measuring temperature and minimize external temperature fluctuations. These additions to the device will greatly depend on the specific characteristics of the place where it will be employed. Second, it is necessary to characterize these sensors with real street samples containing different xylazine and fentanyl concentrations. Considering this, we propose the use of these sensors as potential candidates in routine drug-checking applications and facilities.

### Dye Loading and Displacement Assay

After evaluating the MIPs’ binding behavior toward xylazine as well as the performance of the thermal readout, a dye displacement assay was prepared and characterized. Methylene blue, as a cationic dye, was selected due to its chemical structure containing amine groups and thioesters, also present in xylazine. A binding isotherm was constructed for MB in the same concentration range as for xylazine (Figure S2a,b). As seen from the results, the MIP displayed a considerably higher dye binding capacity (*S*_b‑dye_) than the NIP. Such a difference is illustrated in Figure S2b, where a picture of the stock solution and the resulting filtrates is presented for both MIP and NIP. To determine whether this enhanced binding capacity could be attributed to differences in the polymer macrostructure rather than the presence of imprinted cavities, we investigated the surface area and pore volume of both polymers using nitrogen physisorption.

Interestingly, both polymers exhibit similar surface areas. According to the BET analysis (see Table S3), the NIP displayed an area of 496 m^2^/g, while the MIP showed a slightly lower value of 481 m^2^/g. These values are notably higher than those typically reported for methacrylic acid–based bulk MIPs, which generally show surface areas just above 300 m^2^/g.^36^ Regarding the pore volume, the NIP displayed a marginally higher value of 1.08 cm^3^/g compared to 0.923 cm^3^/g for the MIP.

These minor differences in surface area and pore volume strongly suggest that the enhanced binding capacity of the MIP arises not from differences in the polymer macrostructure but from specific interactions within the imprinted cavities. More specifically, a strong electrostatic interaction between the anionic phosphonic acid groups and the cationic dye.^37^

Next, the thermal stability of the MB-loaded MIPs was evaluated using TGA. The materials displayed high thermal stability under normal environmental conditions, with a degradation onset of 298 °C. This sensor’s property is crucial to ensure reliable results even after being stored at high temperatures.^38^

The displacement assay was evaluated with MB-loaded MIPs and using xylazine hydrochloride aqueous solutions at different concentrations. The concentrations were selected between the maximum and minimum reported for xylazine adulterated fentanyl street samples and ranged from (36–580 μg/mL) (see Table S1).^39^ Parameters such as the amount of powder, incubation time, and solution volume were optimized (not reported here) tobe 4 mg, 5 min, and 2 mL. The dye displacement dose–response curve is presented in Figure 3. The amount of dye displaced was directly proportional to the concentration of xylazine and displayed linear behavior. The color change was observable at concentrations >70 μg/mL. Interestingly, the average xylazine concentration reported for street samples is 440 μg/mL (Table S1); at this value, our sensor response is a strong blue color. Moreover, the assay exhibited a LoD by the naked eye of 76 μg/mL, which is close to the one reported for commercially available lateral flow assays (2 μg/mL).^40^ These results highlight the potential of the dye displacement assay to be used for the detection of xylazine in rapid tests and are comparable to those obtained with antibodies.

**Figure 3:**
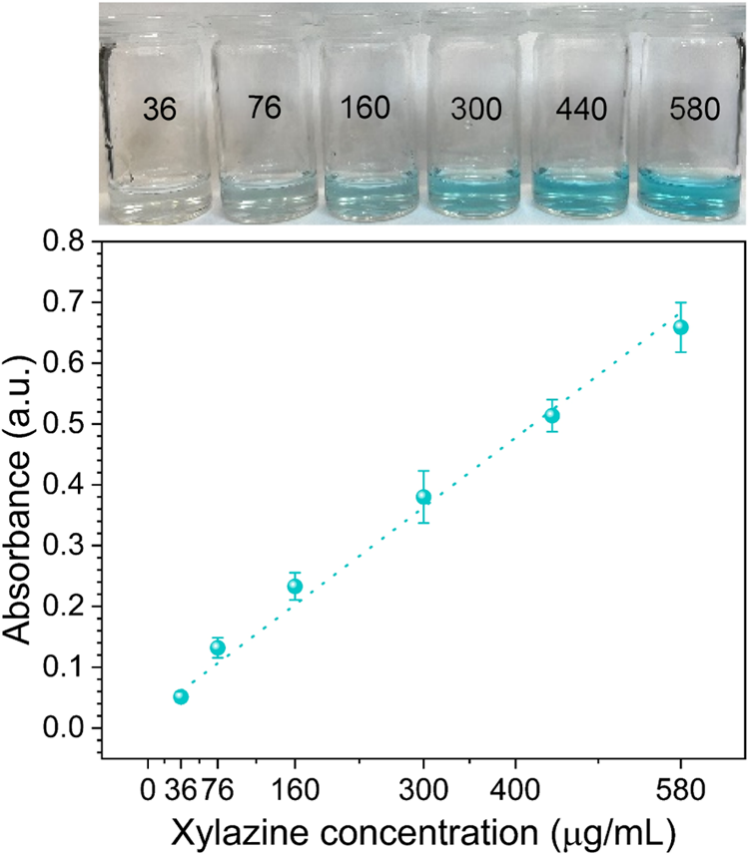
Dye displacement xylazine dose–response curve in D.I water.

### Dye Displacement of Analogue Structures

The cross-reactivity of the dye displacement against xylazine analogs was evaluated at a concentration of 440 μg/mL. For all three compounds, a visual dye release was observed. In the case of tizanidine and clonidine, the response is slightly lower (∼0.4) than that for xylazine (0.5). However, *p*-iodo-clonidine displayed twice the absorbance (∼1) when compared to that of xylazine (Figure 4). The latter can be attributed to the reported MB strong affinity toward iodine species.^41^ Moreover, all the compounds display toxicity similar to that of xylazine, which also poses a health risk if consumed inadvertently at high concentrations. Therefore, due to the highly changing drug supply, the previous results expand the usability of our sensor to detect analogues that can also be used as adulterants in the future.

**Figure 4:**
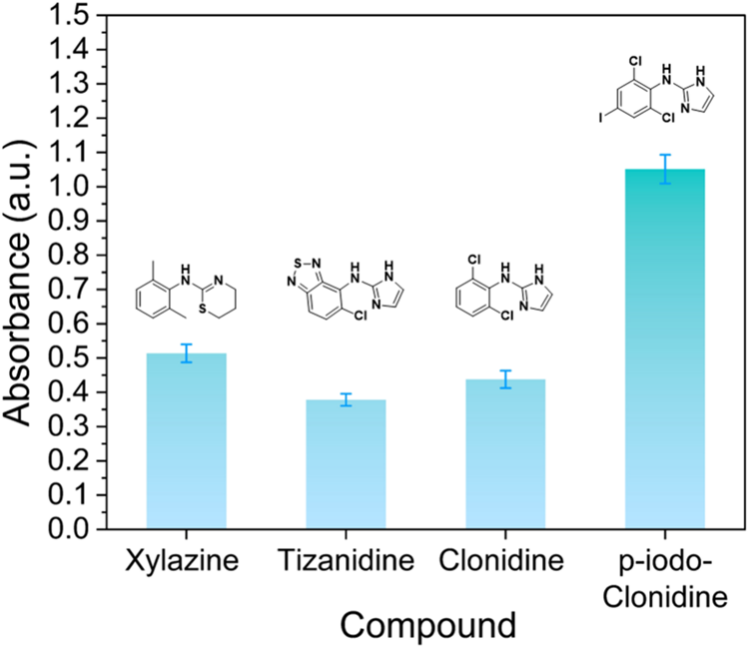
Dye displacement selectivity assay at a concentration of 440 μg/mL.

### Dye Displacement Interference

As briefly mentioned before, one of the challenges in analyzing xylazine in complex drug samples is the heterogeneity of the same. On average, fentanyl samples containing xylazine also contained caffeine, lidocaine, and bulking agents such as mannitol.^39^ Therefore, it is crucial to evaluate the displacement assay in the presence of such compounds at the relevant concentration. In this case, we evaluated all three at the maximum concentration reported in real samples (Table S2). As seen from the results (Figure 5), mannitol and caffeine showed the lowest absorbance value and did not yield a color that could be detected by the naked eye. On the other hand, lidocaine displayed a much higher response than the previous ones (0.18) and could be detected by eye. Lidocaine ability to displace the dye is strongly related to the structural similarities with xylazine as highlighted in the pink circle. Despite this color being detected by the naked eye, the average concentration of lidocaine is almost 10 times lower than the one presented here. These results indicate that the test has high tolerance for the main interference species present in street samples, which decreases the probability of false-positive results.

**Figure 5:**
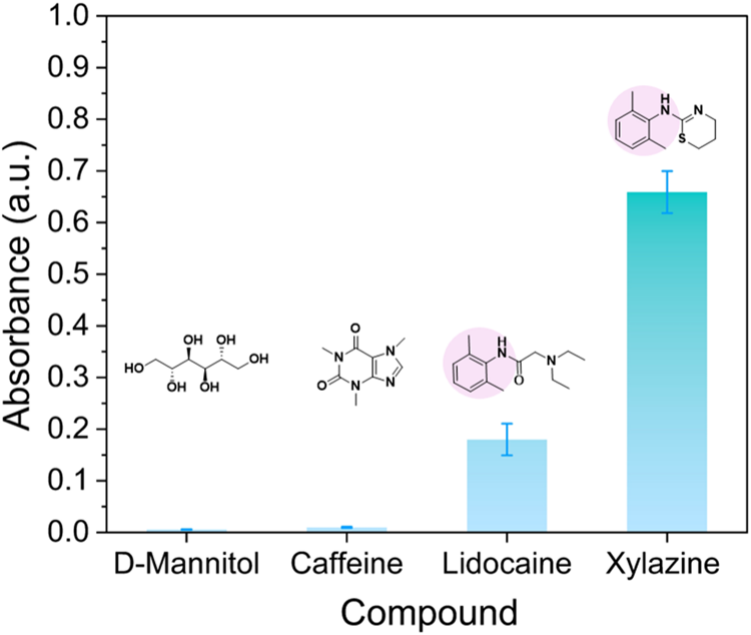
Effect of interferants in the dye displacement assay, interferants were evaluated at the maximum concentration reported, whereas xylazine at 580 μg/mL (see Table S1).

### Real Sample Analysis

The final step was to characterize the sensor in a matrix that closely resembled a real sample. For doing so, we prepared a street-like mixture containing the three interferants (caffeine, lidocaine, and mannitol) together with fentanyl and xylazine (for concentrations see Table S2). A control sample was analyzed in the absence of xylazine. Both sets of results were normalized to account for the effect of pH, NaCl, and citrate anion present in the matrix (see experimental details). As shown in the normalized results (see Figure 6), the fentanyl-containing mixture alone displayed an absorbance value of ∼1, while the xylazine-laced mixture showed a stronger response (∼1.4). Both values are higher than those observed in the distilled water-based tests (see Figures 3–5), which we attribute to the high complexity of the matrix, including a higher pH (∼7.4) and the addition of other interferants such as NaCl and NaOH. This highlights the challenges of the normalization step as it is constrained by the limited information on the fentanyl citrate supplier. Despite this, the sample containing xylazine displayed a significantly higher absorbance (>0.4) in comparison to the one without xylazine. To circumvent the artifacts generated by a complex matrix, we propose the sensor to be used only with deionized/distilled water at pH values around 6–7. Additionally, colorimetric baseline correction can be performed using pure fentanyl at different concentrations.

**Figure 6:**
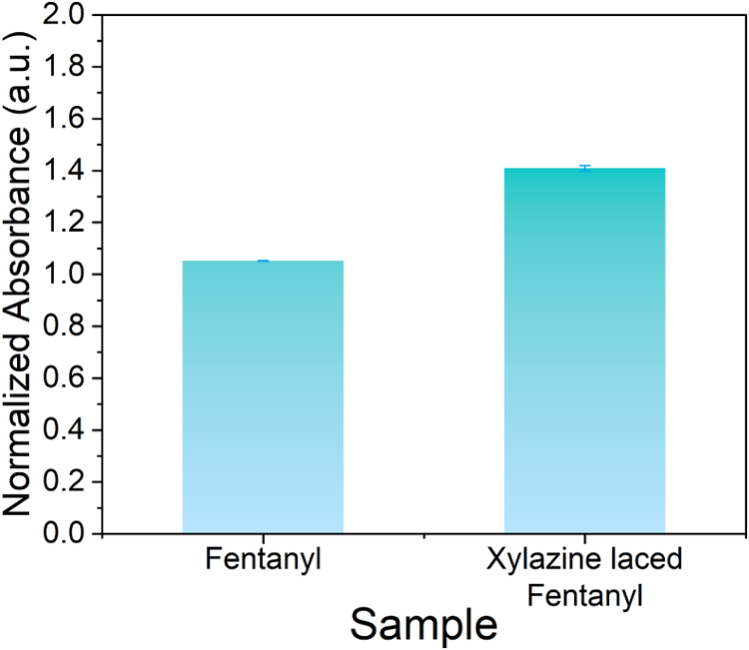
Normalized dye displacement absorbance for samples containing fentanyl and fentanyl-xylazine. Both mixtures contained caffeine, lidocaine, and mannitol (Table S2). Absorbance values are normalized to account for the matrix effect (pH: 7.4, NaCl: 3.5 mg/mL, and citrate monobasic: 50 μg/mL).

The results presented in this section highlight the potential of this sensor for the detection of xylazine in complex fentanyl mixtures with high tolerance to multiple possible interferents under laboratory conditions. However, validation of such a sensor using real environments and street samples is necessary to evaluate its practical applicability. This approach would allow correction for matrix effects and enhance the assay’s practical applicability in real-world scenarios.

## Conclusions and Recommendations

This work highlights the potential of polyvinyl phosphonic acid–based MIPs for the detection of xylazine in two drug-checking scenarios. First, a sensing platform based on HTM enables the detection of low xylazine concentrations (75 nM; 16.5 ng/mL) within a short time per concentration (15 min), making it suitable for use in centralized drug-checking facilities. Second, a colorimetric assay based on dye displacement for the rapid and on-site detection of xylazine at higher concentrations (>76 μg/mL) in under 5 min. Additionally, the sensor was also able to detect the presence of various unwanted xylazine analogues, which can act as potential fentanyl adulterants in the future, adding to the adaptability of the technology in a continuously changing illegal drug supply. Most importantly, the colorimetric sensor was able to detect the presence of xylazine in a highly complex fentanyl mixture containing various possible interferents. As an outlook and improvement aspect for this test, we recommend the use of deionized or distilled water to avoid ionic interferents and a possible false positive result.

Together, the complementary approaches presented in this study highlight how a single MIP formulation can address multiple detection needs, providing an alternative to the existing harm reduction strategies implemented to curb the xylazine-fentanyl epidemic.

## Supplementary Material



## Notes

MIP FTIR spectra colored bands representing the main functional groups (Figure S1); dye binding isotherm extracted from the UV–vis data (A) and visualcomparison of MB stock solutions and their respective filtrate solutions (MIP-NIP) after30 min incubation (B) (Figure S2); descriptive statistics for drug amount based on suspected content (Table S1); real sample concentration conditions (Table S2); nitrogen physisorption results, BET surface area, and BJH pore volume (Table S3) (PDF)
