# Supplementary material for: Drug-Checking with Molecularly Imprinted Polymers: Addressing Xylazine-Contaminated Fentanyl
Source: ACS Sens. 2025 Aug 18;10(11):8458–65. doi: 10.1021/acssensors.5c01931 (PMC12670990; doi:10.1021/acssensors.5c01931)
Supplement: Supplementary file 1 [file se5c01931_si_001.pdf]

Supportive Information

Drug-Checking with Molecularly Imprinted Polymers: Addressing Xylazine-contaminated Fentanyl.

Ramiro Marroquin-Garcia*[1], Gil van Wissen[1], Rocio Arreguin-Campos[1], Gabriela Goraşov[1], Rob van Osch[2], Thomas. J. Cleij[1], Kasper Eersels[1], Bart van Grinsven[1], and Hanne Diliën[1].

[1]Sensor Engineering Department, Faculty of Science and Engineering, Maastricht University, P.O. Box 616, 6200 MD Maastricht, The Netherlands
[2]Zuyderland Medical Center, 6419 PC, Heerlen, The Netherlands

Contents.

1) **Figure S1**. MIP FTIR spectra colored bands representing the main functional groups
2) **Figure S2**. Dye binding isotherm extracted from the UV-Vis data (A) and visual comparison of MB stock solutions and their respective filtrate solutions (MIP-NIP) after 30 min incubation (B).
3) **Table S1.** Descriptive statistics for drug amount based on suspected content (adapted from [1])
4) **Table S2.** Real sample concentration conditions (adapted from [1])
5) **Table S3.** Nitrogen physisorption results, BET surface area, and BJH pore volume.

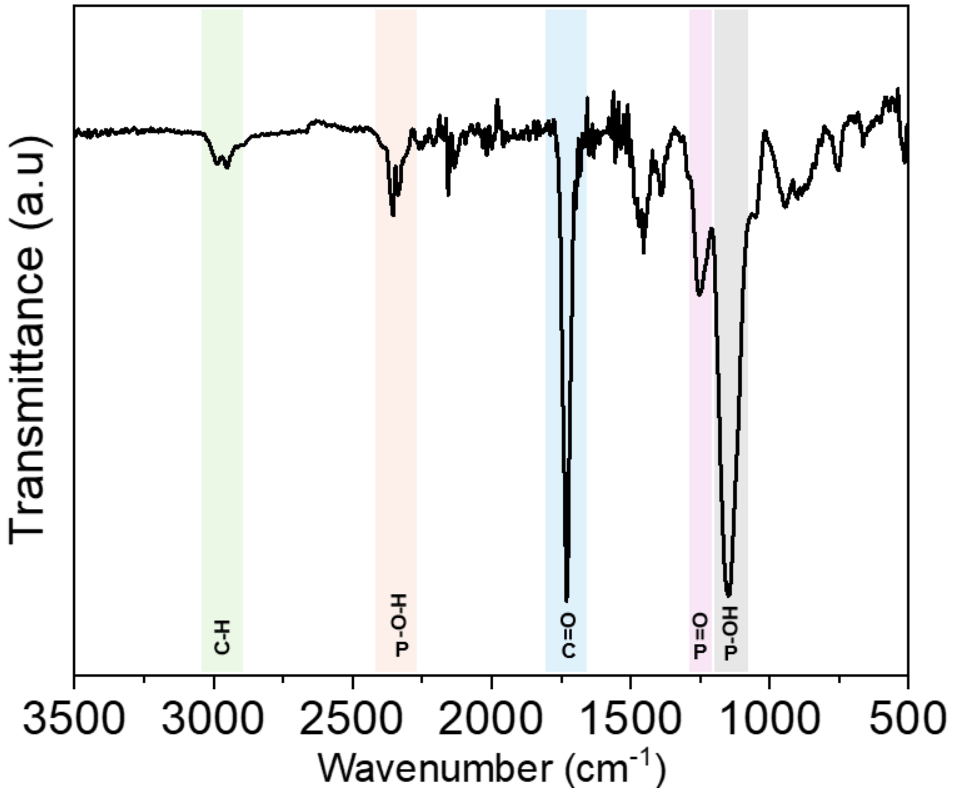

Figure S1. MIP FTIR spectra colored bands representing main functional groups

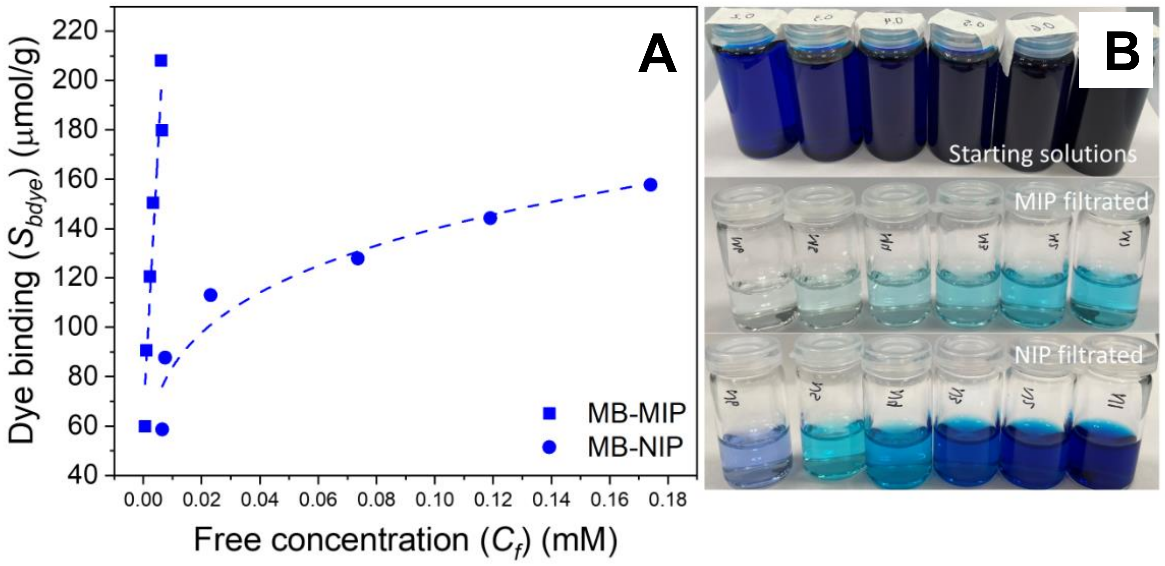

Figure S2. Dye binding isotherm extracted from the UV-Vis data (A) and visual comparison of MB stock solutions and their respective filtrate solutions (MIP-NIP) after 30 min incubation (B).

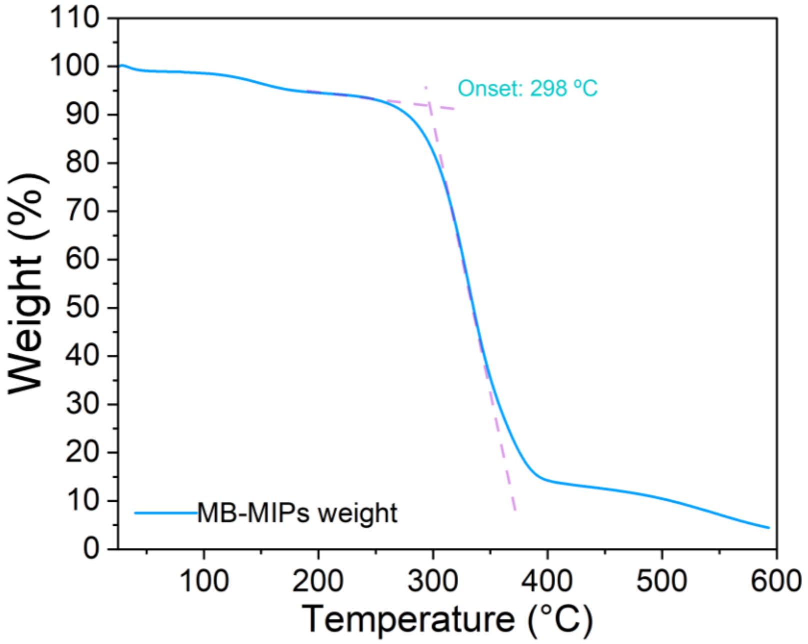

Figure S3. Thermogravimetric analysis (TGA) traces of MB-loaded MIPs under nitrogen atmosphere and with a heating rate of 10 ºC/min. Degradation onset of 298 ºC was calculated using TRIOS software (TA Instruments).

Table S1. Descriptive statistics for drug amount based on suspected content (adapted from [1])

Drug	N	Mean	Median	Min.	Max.	Mean Weight (mg)*	Mean Conc. (mg/mL) **	Min Conc. 1**	Max Conc. 1**
Xylazine	177	44.2 %	45.1 %	0.9 %	71.8 %	2.21	442	-	-
Fentanyl	177	14 %	12.4 %	0.2 %	40 %	0.7	140	-	-
Caffeine	39	4.2 %	1.1 %	0.1 %	23.5 %	0.21	42	-	235
Lidocaine	17	2.8 %	0.8 %	0.2 %	19 %	0.14	28	-	190
Other (Mannitol)	-	34.8 %	-	-	-	1.74	348	-	348

[1] mg/mL
*Assuming 5 mg total weight [1]
** Assuming 5 mL of solvent [2]

Table S2. Real sample concentration conditions (adapted from [1])

Drug	Composition	Weight (mg)*	Conc. (mg/mL) **
Xylazine	44.2 %	2.21	442
Fentanyl	4.97 %	0.25	50
Caffeine	4.2 %	0.21	42
Lidocaine	2.8 %	0.14	28
Other (Mannitol)	34.8 %	1.74	348

*Assuming 5 mg total weight [1]
** Assuming 5 mL of solvent [2]

Table S3. Nitrogen physisorption results, BET surface area, and BJH pore volume.

Sample	BET surface area (m^2/g)	BJH pore volume (cm^3/g)
MIP	481	0.923
NIP	496	1.080

Number of xylazine molecules calculation

1) From the binding isotherm at the high concentration range (Figure 1), we can estimate a binding capacity S_b ~15 μmol/g of MIP after being incubated with a 100 μM solution for 30 minutes at 20 ºC.
2) We can then use the quantified average MIP mass (1 mg) immobilized onto the carbon tape of area =1cm^2
3) However, according to a previous report[1], for a functional layer of 1cm^2, only 65 % of it is in contact with the solution inside the HTM flowcell.
4) Consequently, only 0.65 mg of MIP powder is in contact with the xylazine solution
5) Then, the number of xylazine molecules bound to 0.65 mg (0.00065 g) of MIP powder can be calculated using a S_b ~15 μmol/g or 0.015 mol/g :

$$xylazine\ molecules = \left(0.015\frac{moles\ of\ xylazine}{g\ of\ MIP}\right)(0.00065\ g\ of\ MIP)\left(6.023\ x10^{23}\frac{xylazine\ molecules}{mol\ of\ xylazine}\right)$$

6) # number of xylazine molecules = 5.87 x 10^{18}

References

(1) Debord, J.; Shinefeld, J.; Russell, R.; Denn, M.; Quinter, A.; Logan, B. K.; Teixeira Da Silva, D.; Krotulski, A. J. *Drug Checking Quarterly Report (Q1 and Q2 2023): Philadelphia, PA, USA*.

(2) Krotulski, A. J.; Shinefeld, J.; DeBord, J.; Teixeira da Silva, D.; Logan, B. K. Evaluation of Xylazine Test Strips (BTNX) For Drug Checking Purposes. *Cent. Forensic Sci. Res. Educ.* **2023**.
